# CUZD1 and Anti-CUZD1 Antibodies as Markers of Cancer and Inflammatory Bowel Diseases

**DOI:** 10.1155/2013/968041

**Published:** 2013-04-22

**Authors:** Christos Liaskos, Eirini I. Rigopoulou, Timoklia Orfanidou, Dimitrios P. Bogdanos, Christos N. Papandreou

**Affiliations:** ^1^Cellular Immunotherapy and Molecular Immunodiagnostics, Institute of Research and Technology Thessaly, 41222 Larissa, Greece; ^2^Department of Medicine, Faculty of Medicine, School of Health Sciences, University of Thessaly, Biopolis, 41110 Larissa, Greece; ^3^Division of Transplantation Immunology and Mucosal Biology, King's College London School of Medicine, King's College Hospital, Denmark Hill Campus, London SE5 9RS, UK; ^4^Department of Medical Oncology, University Hospital of Larissa, Faculty of Medicine, School of Health Sciences, University of Thessaly, Biopolis, 41110 Larissa, Greece

## Abstract

CUZD1, the CUB, and zona pellucida-like domains-containing protein 1, is a newly identified antigen of pancreatic autoantibodies (PAB) giving a reticulogranular pattern in patients with inflammatory bowel diseases, and in particular Crohn's disease. The exact mechanisms by which this pancreatic antigen becomes the target of IBD-specific pancreatic autoantibodies are unclear. At the same time, evolving data strongly support a role for CUZD1 in carcinogenesis. Human *CUZD1* is mapped at chromosome 10q26.13 and the loss of this region is a frequent event in various malignant tumours. mRNA overexpression of CUZD1 has been noted in ovarian cancer and serum levels of CUZD1 are elevated in women with ovarian cancer and patients suffering from pancreatic cancer. CUZD1 appears to be one of the relatively few biomarkers that serve as both cancer biomarker and autoantigen of autoantibodies in an autoimmune disease unrelated to cancerous organs. This review discusses the role of CUZD1 in cancer and autoimmunity. We anticipate that a better understanding of the function of CUZD1 will help us to understand how it becomes the focus of an autoimmune attack specifically targeting the intestine and its enigmatic role in carcinogenesis.

## 1. Introduction

In recent years, the identification and validation of novel biomarkers has become the focus of intense research in both the laboratory and clinic. Biomarkers now have several applications and their role has been extended from “diagnostics,” to include “prognostics” and more recently “theranostics.” Theranostics describes a wide range of applications including the identification of a novel diagnostic marker that is used in order to identify patients for whom a newly developed drug will work.

## 2. Cancer Biomarkers and Autoantibody Markers

Cancer biomarkers have changed the way we detect and treat tumors. In recent years, the elucidation of several carcinogenic pathways, and a better understanding of tumor progression, has led to the identification of numerous tumor markers. The list of markers that have been identified is extensive and includes secreted proteins, transcription factors, and cell surface receptors. For example, *α*-fetoprotein (AFP), a member of the albuminoid superfamily, is a cancer biomarker used for the monitoring of hepatoblastoma and hepatocellular carcinoma, as well as certain gastrointestinal cancers [1, 2]. However, an increase of AFP is not always pathognomonic for liver cancer, as elevated AFP levels can be found in end-stage liver disease (cirrhosis) unrelated to tumor development [2, 3]. In fact, virtually all cancer biomarkers lack specificity to a particular tumor type and may be found in a variety of cancerous and noncancerous conditions. Hence, tumor markers alone are not diagnostic for cancer, and at present relatively few tumor markers are widely used by practicing physicians.

Autoantibodies targeting cellular constituents that act as autoantigens are useful serological markers for the diagnosis of autoimmune diseases and are also used in aiding cancer diagnosis [4–7]. Most of them are used for diagnostic purposes, and several of them show very good sensitivities and specificities for particular autoimmune diseases [4, 8–14]. Autoantibodies may also have prognostic significance, being able to identify individuals that will develop overt disease or patients at advanced stages of the disease and also those who will progress in a fast pace. The presence of autoantibodies may also identify patients who may have a better or poorer response to treatment and may also be used to monitor treatment response.

The great majority of tumor markers are not potent autoantibody markers and *vice versa* [15]. However, several studies have addressed the role of autoantibodies and, in particular, tumor-associated antigens (TAA), as targets of humoral and cellular immune responses [15–20]. Various autoantibody specificities have been described in patients with malignancy and some of those appear to be of diagnostic and prognostic significance, being able to allow early diagnosis or stratification of patients according to clinical phenotypes and disease outcome [18]. Investigations are being carried out for the delineation of autoantibody markers useful for the identification of individuals at risk for cancer [20]. Of particular interest are autoantibody markers of paraneoplastic neurological disorders characterised by highly specific autoantibodies directed against onconeuronal antigens [21–27]. Autoantibody panels and signature profiling with diagnostic or prognostic significance for various malignancies are under validation and may prove to be useful in the clinical setting [28]. The prevailing notion has been that antigens overexpressed in a state of a tumour act as cryptic antigens or neoantigens that are perceived as foreign from the immune system [15, 18]. TAA are therefore capable of priming the immune system to recognize TAA and indeed tumor cells expressing them [15, 18]. On the other hand in conditions such as lymphomas, the degenerated B cells produce large amounts of autoantibodies that are insufficiently controlled by the peripheral regulatory machinery of the immune system.

The magnitude, duration, and efficacy of the TAA-specific CD4 and CD8 immune responses depend on several intrinsic factors [29]. An increasing number of studies are investigating ways to manipulate the efficacy of antigen-specific immune responses in a manner that can facilitate the eradication of antigen-expressing tissue-specific target cells [29].

The investigation of the fine specificity of the immune responses against specific TAA has led to the appreciation that some autoantibody specificities related to TAA may bear diagnostic and prognostic significance [17, 20]. An increasing number of studies have obtained data to suggest that several TAA can be potential immunotherapeutic targets, in addition to aiding in the monitoring of disease progression and response to treatment [29–36]. There is no doubt that the study of humoral autoreactivity to TAA has helped investigators to estimate the sensitivity and specificity of individual anti-TAA antibodies. The role of TAA autoantibodies within the processes of carcinogenesis has been a topic of ongoing research. A systematic search of the literature published in 2009 has revealed more than 107 different TAA identified so far, most of which are of limited diagnostic relevance [37]. The majority of these TAA correspond to mutated or overexpressed antigens and were cytoplasmic proteins (42%), while 26% corresponded to nuclear antigens and 21% to membrane-bound proteins [37]. Of interest, only 10% of the reported TAA corresponded to extracellular proteins such as secreted or extracellular matrix proteins [37].

In this review, we discuss the clinical significance of CUZD1, a novel biomarker with a dual role as a cancer and an autoantibody marker.

## 3. CUZD1: Introduction to the Gene

### 3.1. Terminology

CUZD1 stands for CUB and zona pellucida-like domains-containing protein 1. The CUZD1 gene is also known as the uterine-ovarian-specific gene 44 (*UO-44*) and the estrogen-regulated gene 1 (*ERG1*).

### 3.2. Genomic Location

Human *CUZD1 *is mapped at chromosome 10q26.13 and contains nine exons. The loss of 10q is a frequent event in the development and progression of various malignant tumours including prostate adenocarcinoma, endometrial cancers, glioblastoma multiforme, and small cell lung cancer [38–42], suggesting the presence of several tumor suppressor genes in this chromosomal region, which are important for suppression of tumorigenesis and cancer progression.

**Figure 1:**
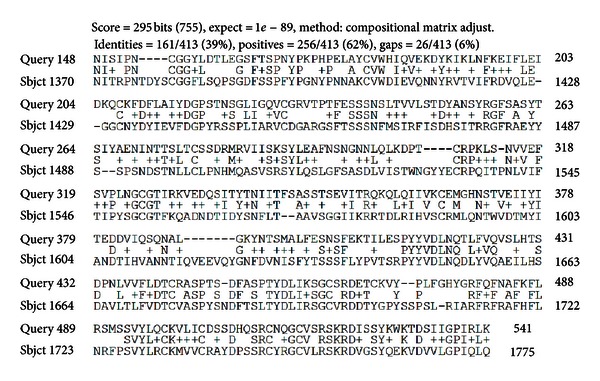
Amino acid comparison between human CUZD1 and DMBT1. There is a high degree of homology between the two proteins possibly reflecting the similarity between the CUB and ZP domains contained in both. The comparison has been performed using the *BLASTp2* protein-protein programme. Top row: CUZD1; bottom row: DMTB1.

The genomic structure of human *CUZD1 *is very similar to parts of the tumor suppressor *DMBT1* (a gene deleted in malignant brain tumors) located at a locus exactly upstream of CUZD1 and in particular at 10q25.3–26.1 [43, 44]. The two proteins share a significant degree of homology (Figure 1).

### 3.3. Structure and Function

**Figure 2:**
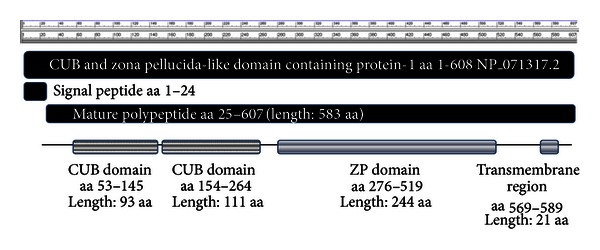
CUZD1 protein features including the CUB and ZP domain exact positioning within the protein.

**Figure 3:**
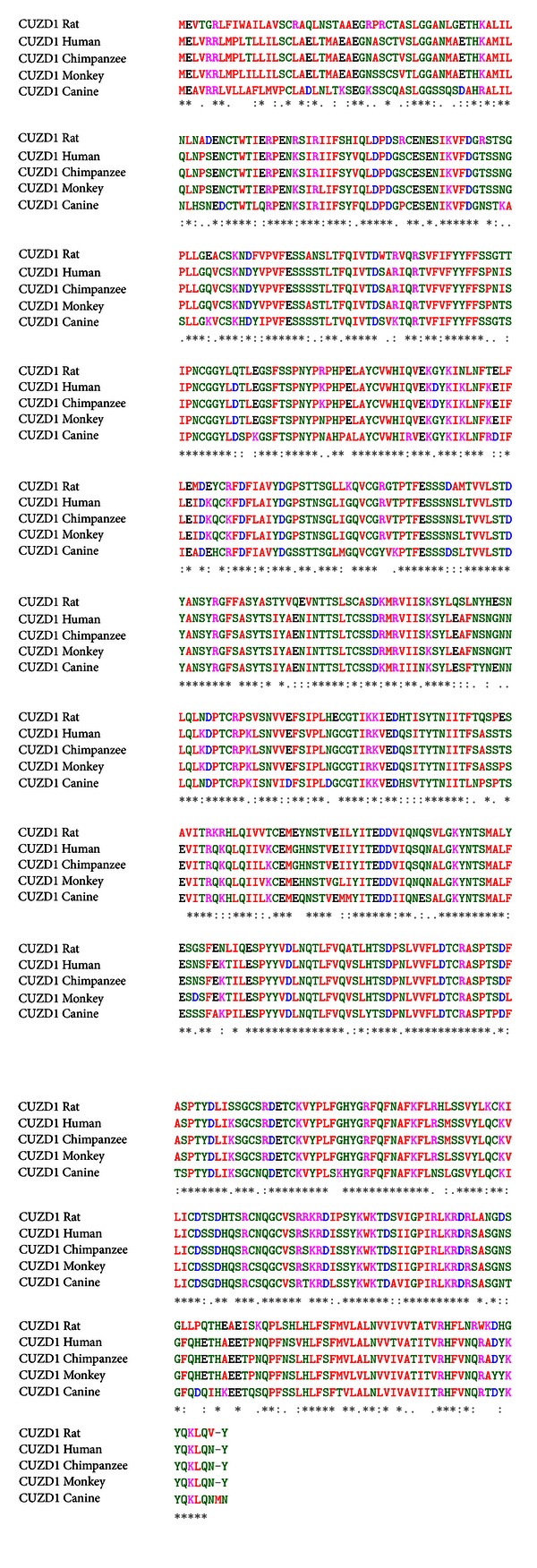
Multiple amino acid sequence alignment of CUZD1 of various species shows striking conservation. Sequences aligned include *Rattus norvegicus* (EDM 11686.1), *Homo sapiens* (NP_071317.2), *Pan troglodytes* (XP_001160209.2), *Macaca fascicularis* (EHH 65083.1), and *Canis lupus familiaris* (XP_544054.2). Asterisk indicates identities and semicolon indicates conserved or semiconserved substitutions. The alignment has been performed using the CLUSTAL W (1.83) multiple sequence alignment tool.

Human *CUZD1* encodes for a 607 amino acids protein with a molecular weight of approximately 68 kDa. CUZD1 (as also the much larger DMBT1) contains two CUB domains and one ZP domain (Figure 2). The protein is highly conserved amongst species (Figure 3).

CUB (complement subcomponents C1r/C1sC1s, Uegf, bone morphogenetic protein) domains [45] are structural motifs frequently present in various extracellular and plasma membrane-associated proteins, many of which are proteases [46, 47]. Each consists of a catalytic domain and several CUB domains. CUB-containing proteins are multifunctional and play a role in embryogenenic signalling [48], complement activation [47, 49], inflammation and autoimmunity [50–52], cell adhesion and motility [53], cell migration and extracellular matrix degradation [54], cell signaling [53, 55, 56], axon guidance and neovascularization [57, 58], neurotransmission, and synaptic plasticity [59, 60]. These proteins have also been involved in fertilization [61], thrombotic microangiopathy [55], cellular uptake and receptor-mediated endocytosis [62], and tumor suppression [51, 53, 63].

ZP glygoproteins (ZP1, ZP2, and ZP3) are responsible for sperm adhesion to the zona pellucida, an extracellular matrix surrounding oocytes [64]. ZP domains have been found in nonmammalian egg-coating multidomain transmembrane proteins such as tumor growth factor-beta receptor III [64, 65], major zymogen granule pancreatic glycoprotein GP2 [66–68], pancreatic ductal protein muclin, uromodulin (also known as Tamm-Horsfall protein) [69], inner ear protein *β*-tectorin, endoglin, no-mechanoreceptor potential-A (NompA), cuticlin-1, and intriguingly, in DMBT1 [70, 71].

### 3.4. Tissue Expression of CUZD1

#### 3.4.1. CUZD1 in Uteri

In 1998, Kasik reported the isolation of a cDNA that was highly expressed in mouse uterus during late pregnancy [72]. At that time, he designated this cDNA as the UTCZP (uterine cub motif zona pellucida) motif [72]. That investigator found that the mRNA encoded by this gene is relatively abundant within the uterus. It first appears 6 days prior to birth and increases over subsequent days to reach maximal levels at 3 days prior to birth. mRNA expression levels then begin to suddenly decrease day by day at the last 3 days of pregnancy, and by the first day following birth it is practically undetectable. Expression of UTCZP mRNA is not found in nonpregnant uterus or in a variety of adult or fetal tissues including fetal and adult brain, thymus, spleen, pregnant maternal liver, fetal liver, adult liver, kidney, heart, and ovary.

In 1999, Chen et al. [73] reported a novel gene exhibiting 82% homology with mouse UTCZP. The expression of this gene induced in the uteri of ovariectomized or immature rats following administration of estrogen and was abolished upon anti-estrogen treatment. These authors termed this gene as estrogen-regulated gene 1 (*ERG1*; GenBank accession number: AF167170). They were able to show that estradiol treatment strongly induces *ERG1* gene in rat uterus and oviduct and that its overexpression is restricted to surface epithelium [73]. The expression level of *ERG1* mRNA is high on day 1 of pregnancy, declined on day 2, and was practically undetectable from days 3 to 6 of gestation. *ERG1* expression was maximal at the proestrus stage of the ovarian cycle, coinciding with the estrogen-induced uterine cell proliferation, and clearly indicating that stage-specific manner of its expression during the ovarian cycle.

Two years later, Huynh et al. [74] reported the isolation of a uterine-ovarian-specific gene 44 (UO-44; GenBank accession number: AF022147) from a tamoxifen-induced rat uterine complementary DNA library in 2001. The UO-44 gene, currently known as *CUZD1*, was specifically expressed in the uterus and ovary of rats [74]. The rat UO-44 cDNA showed 99% homology with the rat *ERG1* cDNAs; thus the two genes were practically identical. In their study, Huynh et al. have shown that UO-44 mRNA was undetectable in uteri derived from OVX rats and was expressed after tamoxifen treatment [74]. In a set of *in situ* hybridization experiments on sections from uteri originated from control OVX and OVX-tamoxifen-treated rats, the same group of investigators have shown that while there was no UO-44 signal in uterus of OVX rats, high levels of UO-44 were detectable in the luminal epithelial cells and glandular population upon treatment with tamoxifen. Intriguingly, treatment with the pure antiestrogen ICI 182780 abrogated the effects of tamoxifen on the expression of UO-44, further suggesting that tamoxifen functions as an estrogen and induces UO-44 expression.

#### 3.4.2. CUZD1 in Ovaries (and in Pancreas)

Huynh et al. have obtained data clearly demonstrating that UO-44 mRNA is detectable in granulosa cells of ovaries [74]. They have also found varying amounts of UO-44 mRNA in granulosa cells of a mixed population of follicles. In particular, high levels of UO-44 expression were noted in the granulosa cells of medium-size follicles, while low-to-moderate UO-44 expression was observed in granulosa cells of small and large follicles. Of interest, the UO-44 mRNA among granulosa cells within the same follicle was not uniform and greatly CUZD1 [75]. In a murine model of necrotizing pancreatitis, UTCZP- (CUZD1-) deficient mice developed more severe pancreatitis suggesting that CUZD1 may play an important role in trypsinogen activation and in the severity of pancreatitis [75].

## 4. CUZD1: As a Cancer Biomarker

One key experiment that suggested an important role for CUZD1 in carcinogenesis demonstrated that human UO-44-specific antisera strikingly inhibit cell attachment and proliferation of NIH-OVCAR3 ovarian cancer cells [76]. This observation has led the authors to postulate that human UO-44 may promote cell growth and facilitate invasion in ovarian cancer. The same group of investigators has shown that cisplatin treatment leads to the downregulation of human UO-44 expression and that silencing of human UO-44 based on sequence-specific siRNAs confers an enhanced sensitivity in cisplatin treatment of human ovarian cancer cells [77]. It also appears that UO-44 in ovarian cancer cells with overexpressed human UO-44 are also resistant to cisplatin [77]. In a recent study, Leung et al. [78] have measured CUZD1 levels in the serum of patients with various types of malignancies and healthy normal controls. Serum samples from patients with ovarian, breast, lung, colorectal, prostate, and testicular cancer were tested. Elevated levels of CUZD1 were found in patients with ovarian cancer, but also in breast and lung cancer [78]. However, this study was conducted in a very small number of sera and the diagnostic significance of CUZD1 as a cancer serum biomarker remains to be assessed in larger cohorts. Nevertheless, the latter study has shown that CUZD1 performed equally well as CA125 in two independent cohorts of samples consisting of healthy controls and ovarian cancer cases, and this may further suggest its potential role as a specific marker of ovarian cancer [78]. The authors reported that CUZD1 is a novel pancreatic cancer serum biomarker as well, but they did not present data to support this statement [78].

## 5. Anti-CUZD1 Antibodies in Inflammatory Bowel Diseases

Patients with Crohn's disease are characterised by the presence of organ-specific and nonorgan-specific autoantibodies [79–89]. Up to 30% of patients with Crohn's disease (CD) contain detectable pancreatic autoantibodies (PAB), giving either a droplet-like or a reticulogranular, cytoplasmic pattern [52, 84–87, 90–93]. The recognition of GP2 as the target of the droplet-like PABs in 2009 [94] has been followed by the recent identification of CUZD1 as the sole autoantigen of PABs giving the reticulogranular, cytoplasmic pattern by indirect immunofluorescence [91–93]. Several studies have attempted to delineate the diagnostic and clinical significance of anti-GP2 PAB [52, 90, 95–99], and the immunopathogenetic role of GP2 in CD has started to emerge [100–102]. However, the biological and clinical significance of humoral and cellular immune responses against CUZD1 remains poorly understood [91–93]. It has become apparent that the two autoreactivities rarely coexist and that the GP2 and CUZD1 are unlikely targets of cross-reactive autoantibodies.

**Figure 4:**
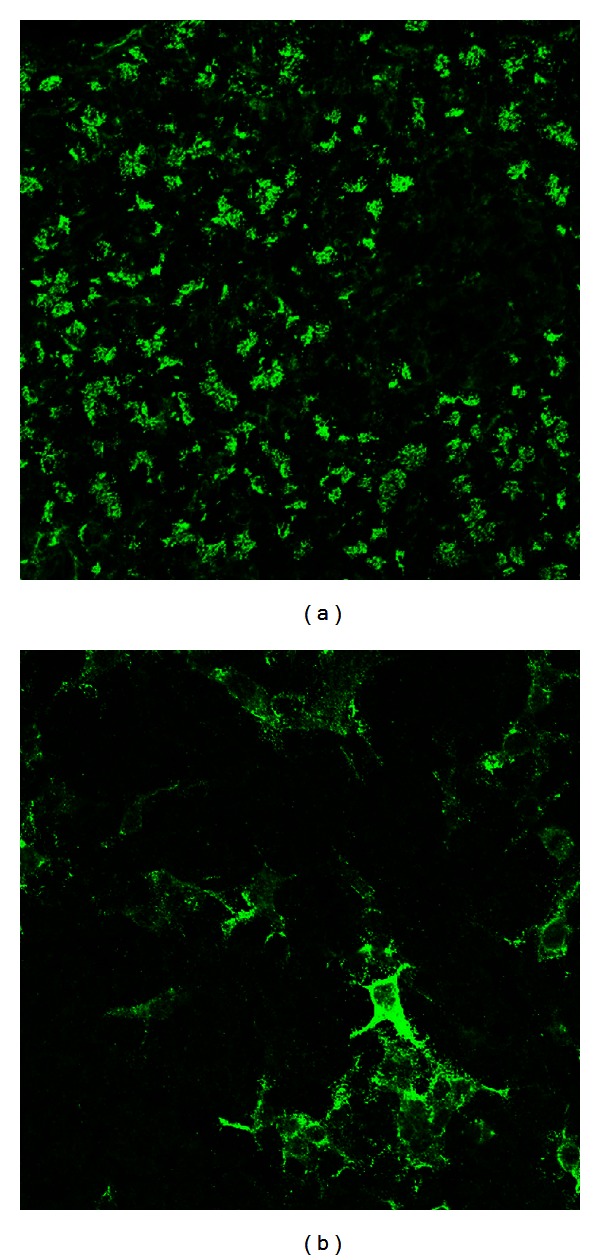
Immunofluorescence staining patterns of CUZD1-specific pancreatic autoantibodies giving a reticulogranular pattern using pancreatic tissue (a) or CUZD1-overexressed HEK3 cells (b).

The group of Winfried Stöcker has identified CUZD1 as the target of PABs giving the reticulogranular, cytoplasmic pattern (Figure 4) [103]. This group has also studied in some detail the diagnostic significance of anti-CUZD1 PABs in inflammatory bowel diseases [103].

### 5.1. CUZD1 as an Autoantigen

The first description of CUZD1 as autoantigenic target has been reported in the form of an abstract in 2008 and more recently as a full paper by Komorowski's group [103]. In a set of experiments, Komorowski et al. showed that the supernatant of homogenized human pancreas completely inhibits PAB reactivity, indicating that the PAB autoantigens are in the soluble fraction [103]. These investigators have tested the ability of various lectins to immobilize PAB-positive glycoproteins from cell-free human pancreas and used one of those, namely, UEA-I (*Ulex europaeus *agglutinin I) to purify the glycoproteins using UEA-I affinity chromatography [103]. GP2 and CUZD1 were identified as the target autoantigens by mass spectrometry. Cloning and eukaryotic expression of CUZD1 has allowed the authors to assess the extent of anti-CUZD1 antibody reactivity using indirect immunofluorescence based on CUZD1 overexpressed human cell line HEK293 [103]. Absorption experiments showed that CUZD1 can completely abolish antibody reactivity of PABs giving the reticulogranular pattern, further indicating that CUZD1 is the only target of PABs giving this pattern [103].

### 5.2. Diagnostic Significance of Anti-CUZD1 Antibodies

Komorowski et al. reported anti-CUZD1 antibodies in 26% of patients with CD (19.8% alone and 6.2% with concomitant anti-GP2 antibodies) [103]. As expected, anti-CUZD1 antibody reactivity was strongly correlated with the reticulogranular PAB pattern.

Kovacs et al. [104] used the same immunofluorescence approach to assess PAB reactivity against CUZD1 and GP2 in pediatric patients with CD. These authors did not provide data for individual reactivity to CUZD1 and GP2, but their overall data indicated the presence of PABs against the two antigens in 35.9% of patients with CD, 24.5% of patients with ulcerative colitis, and in none of the pediatric controls. The unexpectedly high prevalence of anti-CUZD1 and anti-GP2 antibodies in patients with ulcerative colitis warrants further investigations, as PABs are only found in less than 8% of patients with this disease.

More recently, Roggenbuck et al. have also assessed the prevalence of anti-CUZD1 antibodies in their cohort and found that these autoantibodies are present in 29.2% of patients with IBD and, in particular, in 22.6% and 14.9% of patients with CD and UC, respectively [105].

Our own preliminary data in a large cohort of patients with IBD tested by the same technique indicates the presence of IgA or IgG anti-CUZD1 in 21.7% CD compared to 10.8% UC patients (Bogdanos et al., unpublished data). 

### 5.3. Clinical Significance of Anti-CUZD1 Antibodies

Currently, there are no data evaluating the clinical relevance of anti-CUZD1 antibodies in patients with inflammatory bowel diseases.

### 5.4. The Role of CUZD1 in the Immunopathogenesis of Crohn's Disease

The exact role of CUZD1 in the processes that take place prior to the induction of immune-mediated intestinal destruction remains elusive. Komorowski et al. [103] speculated that CUZD1 is released from the pancreas and participates in the innate immune responses that affect the intestinal lumen. The involvement of the ZP domain of CUZD1 may be crucial for the development of intestinal damage, as ZP domains polymerize under specific environmental conditions leading to the aggregation of bacteria preventing their adhesion to mucosal cells and CUZD1 autoantibodies may interfere with such a process [103]. The *de novo* induction of anti-CUZD1 antibodies can be the end result of microbial-induced autoimmunity, similar to that possibly involved in the induction of GP2 autoantibodies [100, 101]. As anti-GP2 and anti-CUZD1 antibodies infrequently cooccur, it may be argued that the mechanisms that lead to the induction of these autoantibodies differ [52].

## 6. Conclusion

CUZD1 is a pancreatic antigen with a dual role, as a cancer biomarker and an autoantibody target. This antigen is one of the very few that may play a role in distinct pathological entities, such as ovarian cancer and Crohn's disease, for reasons poorly understood. The involvement of CUZD1 in carcinogenesis warrants further investigation. We also have to understand the role of CUZD1 in the innate and adaptive immune responses characteristic of CD [52]. We anticipate that the pathogenic role of this antigen will be delineated in the years to come.

## Abbreviations

AFP: *α*-fetoprotein
CUB: Complement subcomponents C1r/C1sC1s, uegf, bone morphogenetic protein
CUZD1: CUB and zona pellucida-like domains-containing protein 1
DMBT1: Deleted in malignant brain tumors
*ERG1*: Estrogen regulated gene 1
IBD: Inflammatory bowel diseases
PAB: Pancreatic autoantibody
TAA: Tumor associated antigens
UO-44: Uterine-ovarian-specific gene 44
UTCZP: UTerine Cub motif Zona Pellucida
ZP: Zona pellucida.
